# Hyperleukocytosis and Leukostasis in Acute Myeloid Leukemia: Can a Better Understanding of the Underlying Molecular Pathophysiology Lead to Novel Treatments?

**DOI:** 10.3390/cells9102310

**Published:** 2020-10-17

**Authors:** Jan Philipp Bewersdorf, Amer M. Zeidan

**Affiliations:** Department of Internal Medicine, Section of Hematology, Yale University School of Medicine, PO Box 208028, New Haven, CT 06520-8028, USA; jan.bewersdorf@yale.edu

**Keywords:** acute myeloid leukemia, AML, hyperleukocytosis, leukostasis, tumor lysis syndrome, disseminated intravascular coagulation, DIC

## Abstract

Up to 18% of patients with acute myeloid leukemia (AML) present with a white blood cell (WBC) count of greater than 100,000/µL, a condition that is frequently referred to as hyperleukocytosis. Hyperleukocytosis has been associated with an adverse prognosis and a higher incidence of life-threatening complications such as leukostasis, disseminated intravascular coagulation (DIC), and tumor lysis syndrome (TLS). The molecular processes underlying hyperleukocytosis have not been fully elucidated yet. However, the interactions between leukemic blasts and endothelial cells leading to leukostasis and DIC as well as the processes in the bone marrow microenvironment leading to the massive entry of leukemic blasts into the peripheral blood are becoming increasingly understood. Leukemic blasts interact with endothelial cells via cell adhesion molecules such as various members of the selectin family which are upregulated via inflammatory cytokines released by leukemic blasts. Besides their role in the development of leukostasis, cell adhesion molecules have also been implicated in leukemic stem cell survival and chemotherapy resistance and can be therapeutically targeted with specific inhibitors such as plerixafor or GMI-1271 (uproleselan). However, in the absence of approved targeted therapies supportive treatment with the uric acid lowering agents allopurinol and rasburicase as well as aggressive intravenous fluid hydration for the treatment and prophylaxis of TLS, transfusion of blood products for the management of DIC, and cytoreduction with intensive chemotherapy, leukapheresis, or hydroxyurea remain the mainstay of therapy for AML patients with hyperleukocytosis.

## 1. Introduction

Acute myeloid leukemia (AML) is characterized by the clonal proliferation of hematopoietic stem cells (HSC) leading to bone marrow failure as well as complications related to the excess production of undifferentiated myeloid blasts such as leukostasis, tumor lysis syndrome (TLS), and disseminated intravascular coagulation (DIC). [1,2] The rates of AML patients who present with hyperleukocytosis, which is commonly defined as a white blood cell (WBC) count of greater than 100,000/µL and has been independently associated with adverse outcomes, are variable among available studies and range from 8–12% in randomized clinical trials to 9–18% in retrospective series [3,4,5,6,7,8]. However, clinical manifestations of leukostasis can already occur with lower WBC counts [9]. Because of early mortality rates as high as 50% during induction therapy or 30 days after presentation, hyperleukocytosis—especially if complicated by leukostasis—is a hematologic emergency that requires prompt treatment with cytoreductive therapies [10,11,12]. While cytoreduction can be achieved using hydroxyurea, intensive chemotherapy, or leukapheresis, the optimal treatment approach is unknown and novel treatments are warranted [9,11,12,13,14].

**Figure 1:**
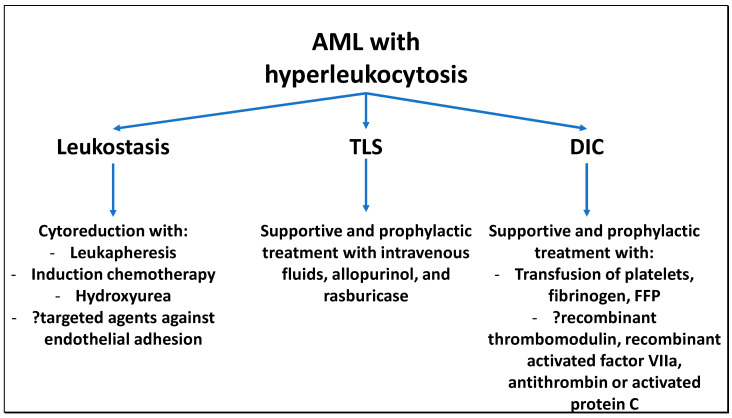
Current and potential future treatment options for complications of hyperleukocytosis in AML: Hyperleukocytosis is associated with a higher rate of leukostasis, tumor lysis syndrome (TLS), and disseminated intravascular coagulation (DIC). Current treatment options for leukostasis include mechanical removal of leukemic blasts with leukapheresis and cytoreduction with chemotherapy or hydroxyurea. As adhesion of leukemic cells to the endothelium is essential to the pathophysiology of leukostasis, targeting blast-endothelial cell interactions might become a future therapeutic strategy. TLS in AML is due to rapid cell turnover leading to electrolyte imbalances and increased serum levels of uric acid that can culminate in renal failure and fatal cardiac arrhythmias. Treatment of TLS entails supportive management of electrolytes, intravenous fluids to maintain urine output, and allopurinol or rasburicase to reduce the production of uric acid. DIC can be managed with transfusion of platelets, fibrinogen, and fresh frozen plasma (FFP). In sepsis-associated DIC as well as in small studies of AML patients, heparin, recombinant thrombomodulin, and other agents have been tested with mixed results and are not part of routine management of DIC associated with AML.

Hyperleukocytosis in AML has been previously associated with various cytogenetic and molecular characteristics. Monocytic and myelomonocytic AML subtypes as defined by the French-American-British (FAB) classification (AML M4 and M5) and patients with *FLT3-*ITD mutations, and abnormalities involving the *MLL* gene on 11q23 have been identified as risk factors for hyperleukocytosis [5,15,16,17]. However, the molecular processes driving the development of hyperleukocytosis are still incompletely understood. Over the past two decades the role of adhesion molecules and cytokines in both the homing of HSCs into the bone marrow and the development of leukostasis has been increasingly characterized [18]. Especially endothelial selectins that promote adherence of leukemic blasts to the vascular endothelium have been identified as essential players not only in the development of leukostasis but also in chemotherapy resistance and leukemic stem cell (LSC) survival [18,19,20,21]. Herein, we provide a brief overview of the interaction of HSCs and LSCs in the bone marrow environment under physiologic circumstances as well as in AML before shifting to the processes in the peripheral circulation leading to DIC, TLS, and leukostasis and a discussion of potential therapeutic targets. Figure 1 provides a schematic illustration of the complications of hyperleukocytosis and currently available and potential future therapeutic approaches.

## 2. The Bone Marrow Niche under Physiologic Conditions and Its Role in the Transformation to AML

The HSC niche in the bone marrow consists of stromal cells, HSCs, and various stages of lineage-specific hematopoietic progenitor cells that interact via complex signaling mechanisms [22,23,24]. In murine models anatomically and functionally distinct niches within the bone marrow have been described [25,26]. Maintenance of HSCs fundamentally depends on the interaction between endothelial and mesenchymal stem cells that regulate HSC quiescence, self-renewal, and proliferation via the secretion of different cytokines such as transforming growth factor (TGF)-β, thrombopoietin, and CXCL12 [24,25]. Those interactions have been reviewed in detail elsewhere recently [24,27,28].

A major limitation to studying the HSC niche is the inability to maintain and expand murine HSC cultures over extended periods of time [29]. Furthermore, there have been discrepant results reported for the use of various Cre-targeted cell lines [24]. Recently, Mende et al. published a computational method to study ligand-receptor interactions between HSCs and other niche cells on a messenger RNA expression level [29]. Using this technique the authors were able to show distinct receptor-ligand interactions between the various cells constituting the bone marrow niche with distinct effects on HSC function that could be altered by the addition of CXCL12 or E-selectin [29]. With the expansion of this technique to single-cell proteomic analyses, an even more granular description of the interactions within the bone marrow niche could be achieved which might allow for the identification of novel therapeutic targets.

The role of the HSC niche in AML development has been speculated for decades [24,28]. Acquisition of mutations leading to functional alterations of niche cells has been suggested based on data showing genetic and epigenetic differences in mesenchymal stem cells from healthy volunteers compared with AML patients [30,31,32]. However, these mutations appear to be distinct from the leukemic driver mutations in leukemic blasts and their functional relevance warrants additional studies [33]. For example, deletion of *Dicer 1*, a gene coding for an RNA-processing endonuclease in bone progenitor cells, resulted in the development of a MDS-like phenotype in mice that occasionally progressed into AML [34]. Similarly, mutations in Notch signaling and *Ptpn11* in bone marrow osteoblasts and mesenchymal cells have been implicated in leukemogenesis [35,36]. Additionally, the contribution of a pro-inflammatory environment in the bone marrow on HSC ageing and the development of myeloproliferative neoplasms and clonal hematopoiesis has been shown [24,37,38]. However, small clonal populations harboring mutations in leukemic driver genes are often present for years and it has been proposed that those clones have a competitive advantage over normal cells leading to the expansion of the cell clone especially after the acquisition of additional mutations [39,40,41]. Additionally, LSCs themselves can induce a leukemia-permissive niche environment [24,28].

**Figure 2:**
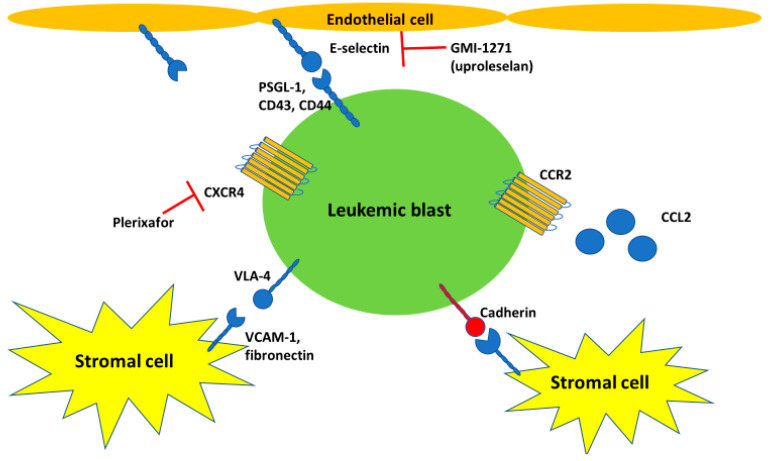
Selected interactions between leukemic blasts and other cells in the bone marrow niche: AML blasts interact via various mechanisms with bone marrow stromal cells and endothelial cells. Among those mechanisms are the interaction of chemokine receptor CXCR4 on leukemic blasts with its soluble ligand CXCL12 (also known as SDF-1), which can be blocked with plerixafor. Additionally, the interaction between E-selectin on endothelial cells and various ligands on leukemic cells such as PSGL-1, CD43, and CD44 has become an increasingly studied therapeutic target with GMI-1271 (uproleselan) currently being studied in advanced phase clinical trials. Other interactions between bone marrow stromal cells and both regular HSCs and leukemic cells include VLA-4/VCAM-1, VLA-5/fibronectin, and cadherins. Finally, the CCL2/CCR2 axis has been shown to be expressed in the majority of monocytoid AML blasts and to play a role in cell proliferation [52].

The mechanisms by which leukemic blast mobilization from and homing to the bone marrow occurs remain incompletely defined. HSCs express and interact with bone marrow stromal cells via various cell adhesion molecules that belong to the integrin receptor superfamily such as VLA-4, VLA-5, and LFA-1 which act as receptors for fibronectin and intracellular adhesion molecule-1 (ICAM-1) [42,43]. Other essential means of interaction between AML blasts and stromal cells are via CXCL12, CD44, selectins, and cadherins [43]. For example, the interaction between VLA-4 and fibronectin is essential for HSC renewal and differentiation and antibodies against VLA-4 have been shown to increase G-CSF-mediated mobilization of HSCs into the peripheral blood [44]. Similarly, targeting CD44 and the CXCL12/CXCR4 axis has been shown in preclinical studies to eliminate leukemic stem cells and to increase the sensitivity to chemotherapy [45,46]. While inhibition of adhesion molecules such as VLA-4, CXCR4, and PSGL-1 can be used to increase the yield of CD34^+^ HSCs during leukapheresis for stem cell transplantation, VLA-4 could also play a role in chemotherapy resistance and LSC survival [42,47]. The interaction between VLA-4 and VCAM-1 protects leukemic blasts from apoptosis and has been linked to minimal residual disease and eventual disease relapse [48]. As a proof of concept, inhibition of VLA-4 has been shown to increase sensitivity to chemotherapy in murine models of acute lymphoblastic leukemia and in vitro models of AML [49,50]. Supporting the role of cellular adhesion molecules in AML are studies showing an interaction between *FLT3-*ITD mutated blasts and VLA-4. In vitro data suggest that upregulation of integrins reduced the sensitivity of *FLT3-*ITD-mutant cells to sorafenib via the phosphatidylinositol 3-kinase (PI3K)/Akt/glycogen synthase kinase-3 beta (GSK3β) pathway leading to enhanced β-catenin activation [51]. Additionally, differences in expression patterns of adhesion molecules between AML blasts and HSCs have been described with VLA-1, -2, -3, and -6, CD31, CD38 being upregulated on AML blasts [43]. Figure 2 illustrates the main interaction between AML blasts and other cells in the bone marrow microenvironment.

Under physiologic conditions mobilization of HSCs is regulated by cytokines, circadian rhythm, and hormonal signals [22,53]. Several hematopoietic cytokines such as G-CSF, KIT ligand, or FLT3 ligand have been shown to induce mobilization of HSCs by suppressing CXCR4 and CXCL12 expression and osteoblast activity [22,54]. As these effects are mediated via monocytic cell populations, this might serve as an explanation for the higher proportion of hyperleukocytosis among patients with monocytic AML [5]. In AML, reduced adhesion of AML blasts has been reported in cells harboring the *RUNX1-RUNX1T1* fusion gene leading to an enhanced migration into the peripheral blood [55]. Furthermore, in a recent retrospective study of 693 de novo AML patients with a WBC count of >50,000/µL had higher incidences of *FLT3-*ITD, *NPM1*, *DNMT3A*, *NRAS, CEBPA,* and *TET2* mutations and the presence of hyperleukocytosis was independently associated with an adverse prognosis [56]. However, the mechanisms by which these mutations lead to hyperleukocytosis remain poorly understood and warrant additional studies. It is also notable that there is an association between various risk factors such as the high prevalence of *NPM1* and *FLT3* mutations and AML M4 and M5 subtypes by FAB classification; all of which have been previously associated with a higher risk of hyperleukocytosis [15,56,57]. Table 1 provides an overview of the various risk factors that been identified as being associated with hyperleukocytosis.

## 3. Interaction between Leukemic Cells and Endothelial Cells and Molecular Processes Underlying Leukostasis

Adhesion of leukocytes to the endothelium is a prerequisite for the recruitment of leukocytes to sites of inflammation and is mediated by the interaction between selectins (L-, P-, and E-selectin) expressed on endothelial cells and leukocytes and integrins on leukocytes as well as several cell adhesion molecules (e.g., CD43, CD44, P-selectin glycoprotein ligand-1 [PSGL-1]) [60,61]. The first step in this process is the activation of endothelial cells by proinflammatory cytokines such as interleukin (IL)-1β and tumor necrosis factor (TNF)-α leading to the increased expression of selectins and integrin ligands [60]. This interaction leads to the slow rolling of leukocytes along the endothelium and allows migration across the vessel wall into the interstitial space as guided by chemokines [60].

Leukostasis has been described as a life-threatening complication of hyperleukocytosis in AML for decades. It has been noted that the extent of hyperleukocytosis in AML does not necessarily correlate with the likelihood of developing leukostasis which suggests specific molecular interactions between AML blasts and endothelial cells leading to leukostasis [9,62,63]. The development of leukostasis in AML has been attributed to a disruption in the microcirculation due to increased blood viscosity as well as a reduced deformability of myeloid blasts compared to both lymphoid blasts and mature myeloid cells [9,64]. However, Lichtman et al. also showed that a reduction in the erythrocrit is able to counterbalance the increase in leukocrit resulting in a normal blood viscosity in patients with hyperleukocytosis [64,65].

Given the high mortality associated with leukostasis and the need for urgent cytoreduction in the absence of a diagnostic gold standard, predictive scores to supplement clinical assessment have been developed [66]. Acknowledging the variable risk of leukostasis among different leukemia subtypes, Novotny et al. proposed a score predicting the likelihood of leukostasis based on various clinical features (primarily neurologic and pulmonary). Patients deemed to have highly probable leukostasis have AML M4/5 and higher WBC and myeloblast counts [66]. However, what specifically leads to leukostasis on a cellular level remains to be fully elucidated.

AML blasts have been shown to express various cell adhesion molecules such as CD11, CD49, PSGL-1, and L-selectin with significant differences between various subtypes of AML blasts [18,19,43,67]. Additionally, leukemic blasts—in contrast to normal neutrophils—have shown to interact with E-selectin via PSGL-1, CD43, and CD44 and to exhibit various expression and activity levels of those selectin ligands that are distinct from normal HSCs and are associated with chemotherapy resistance [18,20]. Leukemic blasts from AML patients have also been demonstrated to secrete IL-1β and TNF-α that lead to the expression of selectins and VCAM on endothelial cells enabling the adhesion of leukemic blasts and could contribute to leukostasis and migration into extramedullary tissues [19,68]. Similar data suggesting endothelial activation have also been published from patients with myelocytic leukemia and pulmonary leukostasis [69]. Additionally, animal studies have suggested a contribution of the complement system to the development of pulmonary leukostasis in AML [70]. The finding by Stucki et al. that anti-TNF-α or anti-IL-1β antibodies were able to prevent endothelial cell activation in vitro raises the option for a novel therapeutic approach to leukostasis [19]. Similarly, antibodies directed against VCAM-1, ICAM-1, E- and P-selectin suppressed endothelial cell activation [19]. Finally, the aberrant expression of CD56/NCAM by leukemic blasts has also been associated with the development of leukostasis in AML patients [71,72].

**Figure 3:**
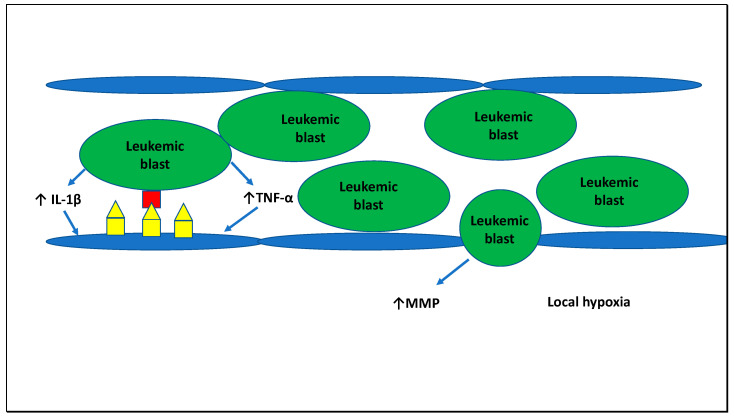
Pathophysiology of leukostasis in AML: Leukostasis in AML is due to various factors. First, myeloblasts are less pliable than mature granulocytes or lymphoblasts and cause mechanical obstruction of small blood vessels leading to hypoperfusion and ischemic damage in distal areas. Second, leukemic blasts produce pro-inflammatory cytokines such as TNF-α or IL-1β that induce the expression of cell adhesion molecules such as E-/P-selectin, ICAM-1, and VCAM on endothelial cells that interact with adhesion molecules on leukemic blasts (L-selectin, CD43, CD44, P-selectin glycoprotein ligand-1 [PSGL-1]). Third, leukemic blasts release matrix metalloproteinases (MMP) that damage endothelial integrity and enable extravasation of leukemic blasts into tissues and can cause microhemorrhages.

Besides adhesion of leukemic blasts to the endothelium, other mechanisms of tissue damage in leukostasis include cytokine- and matrix-metalloproteinase (MMP)-mediated endothelial damage, leukemic blast extravasation into tissues and local hypoxia among others (Figure 3) [9,73,74,75]. The MMP-mediated loss of endothelial integrity has been associated with extravasation of leukemic blasts into the interstitial space as well as microhemorrhages [9,74]. Additionally, both the mechanical obstruction and the increased metabolic demand in the microvasculature have been proposed on leading to hypoxic injury on a local tissue level [9]. However, the contribution of these mechanisms to the development of leukostasis and their role as a potential therapeutic target require additional studies.

## 4. Coagulopathy

**Figure 4:**
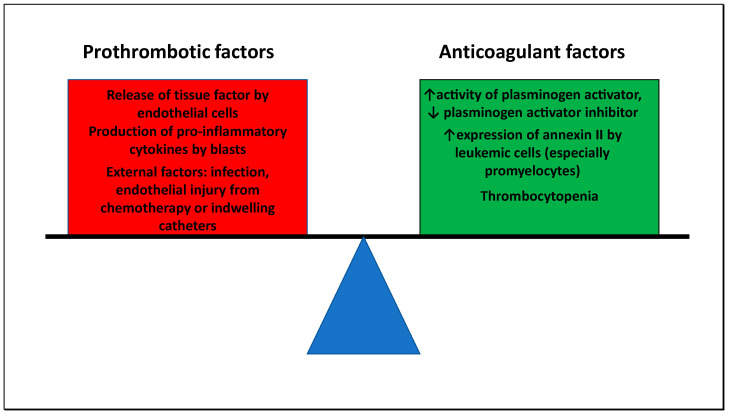
Pathophysiology of disseminated intravascular coagulation: DIC is characterized by an imbalance of pro- and anticoagulant factors due to both excess activation of the coagulation system and increased fibrinolysis. Prothrombotic factors in AML include the release of tissue factor from endothelial cells which is at least partly stimulated by the production of pro-inflammatory cytokines by leukemic blasts as well as external factors that promote endothelial injury such as infections, chemotherapy, or indwelling catheters. Simultaneously, anticoagulant factors contributing to the development of DIC include increased activity of plasminogen activator in conjunction with reduced levels of plasminogen activator inhibitor, increased expression of annexin II by leukemic cells, and disease-related thrombocytopenia.

Overt disseminated intravascular coagulation (DIC) has been reported in up to 32% of patients with non-APL AML [76,77,78]. DIC is characterized by both excess activation of the coagulation system due to the release of tissue factor from endothelial cells and leukemic cells (especially promyelocytes) and increased fibrinolysis in the setting of elevated plasminogen activator and expression of annexin II [79,80,81]. Laboratory abnormalities consistent with DIC (decreased fibrinogen and platelets, elevated d-dimer, prolonged prothrombin and activated partial thromboplastin time) have been documented in 30–40% of AML patients presenting with hyperleukocytosis although the diagnostic value of a low platelet count in this setting is questionable as it is more likely related to the underlying disease rather than a consumptive process [9,82]. In patients presenting with WBC counts >100,000/µL rates of DIC of 63% have been reported [77]. The high rate of DIC in hyperleukocytosis is an additional challenge in patients with leukostasis as leukapheresis can potentially worsen coagulopathy [83]. Increased exposure of tissue factor by endothelial cells has also been linked to the release of pro-inflammatory cytokines such as IL-1 from apoptotic leukemic blasts, which potentially explains the high rates of DIC seen in patients with hyperleukocytosis given the rapid cell turnover [79,84]. Furthermore, external factors such as infection and vascular endothelial injury due to leukemia-directed therapy are likely contributing as well. It is important to emphasize that while patients with DIC are prone to hemorrhagic complications, a recent prospective cohort study of newly diagnosed AML patients treated with intensive chemotherapy also showed rates of venous and arterial thrombosis of 8–10% with significantly higher rates seen in patients with laboratory evidence of DIC and especially an elevated d-dimer prior to initiation of therapy [76]. Figure 4 depicts the pathophysiology of DIC in patients with AML.

Risk factors for the development of DIC in non-APL AML patients include an elevated WBC count, high levels of C-reactive protein, CD13- and HLA-DR-negativity, and 11q23 abnormalities [76,77]. Because of an increased risk for both thrombotic and hemorrhagic complications, treatment of DIC is challenging with transfusion of platelets, fibrinogen, or fresh frozen plasma constituting the mainstay of therapy [9]. Recently, recombinant soluble thrombomodulin (ART-123), an activator of the intrinsic anticoagulant protein C that is approved for treatment of DIC in Japan, has been shown to be superior compared to heparin in a randomized trial in DIC patients (32% non-APL AML patients) although results were not reported separately for AML patients [85]. Similar results have been reported from additional single-arm studies in AML [86,87]. Other alternative agents that have been tested for the management of DIC include recombinant activated factor VIIa, antithrombin, or activated protein C [88]. However, the currently available evidence supporting the use of those agents is very limited and none of these agents are currently undergoing advanced phases of clinical testing [88]. Additional, ideally randomized trials are necessary to evaluate the safety and efficacy of those agents and to assess their effect on survival outcomes.

## 5. Tumor Lysis Syndrome

AML patients with hyperleukocytosis are at risk for spontaneous TLS and even more so after the initiation of cytoreductive therapy. TLS is characterized by hyperuricemia, hyperphosphatemia, hypocalcemia, and hyperkalemia because of the rapid destruction of tumor cells which can be complicated by renal failure due to urate crystal deposition in the kidneys and cardiac arrhythmias [89]. Based on the Cairo-Bishop classification, TLS is classified as either laboratory or clinical TLS [90]. Compared with lymphoid malignancies like aggressive lymphomas and acute lymphoblastic leukemia, spontaneous TLS is rare in AML with poorly defined triggers that are mostly extrapolated from patients with lymphoid malignancies such as the rapid proliferation of malignant cells leading to cell lysis [91].

Reports on the incidence of TLS in AML patients vary substantially because of the various definitions and implementation of preventive measures with rates of TLS of 3–17% having been reported [91,92,93]. However, the risk of TLS in AML patients with hyperleukocytosis can be substantially higher with rates of 26–50% and 45% having been reported in patients with a WBC of >50,000/µL and >100,000/µL at presentation, respectively, in several retrospective studies [11,13,92,94]. In a retrospective study of 772 AML patients undergoing induction chemotherapy for AML, elevated lactate dehydrogenase, uric acid > 7.5 mg/dL, creatinine > 1.4 mg/dL, and WBC > 25,000/µL had been identified as risk factors for the development of TLS. As expected, the risk of TLS increased with the degree of WBC elevation with patients presenting with a WBC > 75,000/µL having a 5.8 (95% CI: 2.2–14.4) and 9.6 (95% CI: 4.4–17.6) higher odds for the development of clinical and laboratory TLS, respectively, compared with patients with a WBC ≤ 25,000/µL [92].

Several scoring systems have been proposed to predict the likelihood of developing TLS in patients receiving induction chemotherapy [92,95]. However, external validation is lacking and none of those is part of routine clinical practice. As preventive strategies such as aggressive hydration, allopurinol or rasburicase, a recombinant version of urate oxidase, have been shown to be effective in reducing morbidity related to TLS, such measures are generally recommended in AML patients presenting with hyperleukocytosis [9,89,96].

## 6. Current and Novel Molecularly Targeted Therapies for Hyperleukocytosis and Leukostasis

Hyperleukocytosis, especially if associated with leukostasis, constitutes a hematologic emergency that warrants urgent cytoreduction. Options for cytoreduction include mechanical removal of leukemic cells via leukapheresis and pharmacologic cytoreduction with hydroxyurea or intensive chemotherapy [9,16]. Because of the rarity of the scenario no randomized clinical trials comparing those modalities have been conducted and the optimal treatment strategy remains unknown. In two recent meta-analyses leukapheresis did not provide a survival benefit which questions the use of this resource-intensive therapeutic modality [12,14]. However, the cytoreductive effect of hydroxyurea may take several days which limits its utility in the setting of acutely life-threatening leukostasis [16]. Additionally, treatment with hydroxyurea was not associated with a mortality benefit in a recent meta-analysis [14]. Given the transient nature of the cytoreduction with both hydroxyurea and leukapheresis, leukemia-directed chemotherapy should be administered to all patients as soon as feasible per European Leukemia Net recommendations [97]. This further highlights the need for novel therapies targeting the underlying molecular processes of leukostasis directly.

Since 2017 the treatment landscape of AML has significantly expanded with the approval of nine novel agents in various settings in the United States [98,99]. Among those are molecularly targeted agents such as the mutant IDH1 and IDH2 inhibitors ivosidenib and enasidenib, the FLT3 inhibitors midostaurin and gilteritinib, the BCL2 inhibitor venetoclax, the anti-CD33 antibody-drug conjugate gemtuzumab ozogamicin, and the hedgehog inhibitor glasdegib that have all been shown to improve survival in clinical trials either as monotherapy or in combination strategies [100,101,102,103,104,105,106,107]. Additionally, the oral hypomethylating agent (HMA) CC-486 and the liposomal formulation of cytarabine and daunorubicin have garnered regulatory approval [108,109]. However, it is important to note that no dedicated clinical trials in AML patients with hyperleukocytosis exist and the proportion of and outcomes among this patient population have not been reported in recent clinical trials, which greatly limits the evidence base in this setting. Furthermore, because of the concerns about tumor-lysis syndrome patients with a WBC ≥ 25,000/µL were excluded from the venetoclax-based clinical trials unless cytoreduction with hydroxyurea or leukapheresis had been successfully performed previously [100,101].Table 2 provides an overview of those landmark trials and highlights the proportion of patients with hyperleukocytosis. Therefore, extrapolation of those results to patients with hyperleukocytosis is limited and warrants additional dedicated studies or subgroup analyses of previously completed trials. This also applies to FLT3 inhibitors although the presence of *FLT3*-ITD mutations has been associated with a higher incidence of hyperleukocytosis and those agents could therefore be a valuable adjunct in those patients [104]. Additionally, treatment with IDH inhibitors can lead to worsening of leukocytosis because of the differentiation syndrome especially during earlier treatment cycles with ≥grade 3 leukocytosis having been reported in 1.7–5% of patients [102,105].

Unfortunately, randomized clinical trials specifically enrolling patients with leukostasis are exceedingly challenging to conduct given the rarity and acuity of the clinical presentation. However, targeting the interaction between LSC and selectins on endothelial cells is an area of active research as upregulation of E-selectin on endothelial cells has been associated with LSC survival and chemotherapy resistance [20,111]. Inhibition of E-selectin with the small molecule GMI-1271 (uproleselan) in combination with chemotherapy has been shown to prolong survival in a mouse model of AML [21]. Mechanistically, Barbier et al. showed in murine experiments using adoptive transfer of HSCs that had been retrovirally transduced with the human *MLL-AF9* fusion oncogene that AML blasts exhibit an increased E-selectin-binding potential that enabled retention of AML blasts within the bone marrow and could contribute to chemotherapy resistance [21]. Furthermore, treatment with GMI-1271 increased mobilization of AML blasts from the bone marrow and sensitized them to the effects of cytarabine with a concurrent reduction in the number of LSCs resulting in a survival advantage compared to control mice [21]. Similar results were achieved in mice with E-selectin gene-deletion (*Sele*^−/−^) further supporting the mechanism of action of GMI-1271 via E-selectin inhibition [21]. Additionally, treatment with GMI-1271 blocks signaling via the PI3K/AKT/NF-kB and RAS/MAPK/ERK pathways; two pathways that have been associated with AML blast survival [21,112]. In a phase I study GMI-1271 has been shown to be safe with infusion site reactions being the most common adverse event and no mobilization of HSCs into the peripheral blood in healthy volunteers [113]. This led to a phase I/II clinical trial of GMI-1271 in combination with mitoxantrone, etoposide, cytarabine (MEC) chemotherapy in relapsed/refractory AML patients or in addition to 7 + 3 induction chemotherapy with cytarabine and idarubicin in newly diagnosed AML patients [114]. With a rate of complete remission (CR)/CR with incomplete cell count recovery (CRi) of 41% and 68% among relapsed/refractory AML patients and treatment-naïve patients, respectively, those results appear promising but need to be verified in the ongoing phase III, placebo-controlled trial (NCT03616470, NCT03701308) [114]. Importantly, the addition of GMI-1271 to standard induction chemotherapy did not result in excess toxicity and 30-day and 60-day mortality rates of 8% and 12%, respectively, appear to be comparable to those observed with 7 + 3 induction in routine clinical practice [114,115]. Similar preclinical and clinical results have been reported for the CXCR4 antagonist plerixafor that has been combined with both intensive chemotherapy and hypomethylating agents with an acceptable safety profile (NCT01352650, NCT01435343) [116,117,118]. However, a major concern with inhibitors of cell adhesion is the potential of worsening hyperleukocytosis as they could lead to increased mobilization of leukemic blasts from the bone marrow. Notably, inhibition of CXCR4 did not lead to hyperleukocytosis in clinical trials [118]. VLA-4 serves as an another therapeutic target with two inhibitors (AS101 and FNIII14) having shown to promote chemotherapy sensitivity in in vitro studies leading to a phase II trial in human AML and MDS patients [50,119]. However, the phase II trial of AS101 has been suspended (NCT01010373). Several other compounds targeting LSC release from the bone marrow niche have been tested and recently reviewed [120]. Although we acknowledge the limitations of extrapolating those results to patients with leukostasis, the central role of selectins in the adherence of leukemic blasts to endothelial cells and the contribution to leukostasis might make such an approach a potentially promising target.

Acute lung injury secondary to leukostasis is a common complication in AML patients presenting with hyperleukocytosis and especially in patients with acute monocytic leukemia (AML FAB-M5) [13,121,122]. In a small, single center, prospective study of 20 treatment-naïve patients with acute monocytic leukemia admitted to an intensive care unit (ICU) in France with acute respiratory failure, treatment with dexamethasone (10 mg every 6 h) in addition to chemotherapy and empiric broad-spectrum antibiotic coverage resulted in significantly lower rates of ICU mortality compared to historic controls (20% vs. 50%; p = 0.04) [122]. The presumed mechanism underlying this mortality benefit is the reduction of post-chemotherapy lysis pneumopathy, leukostasis, and leukemic infiltration [122]. Reassuringly, no increase in bacterial or fungal infections was seen in dexamethasone-treated patients [122]. Additionally, a recent study in an AML rat model suggested that dexamethasone does not reduce the anti-leukemic effects of cytarabine unlike prior studies that suggested that dexamethasone inhibits deoxycytidine kinase, the enzyme that is responsible for activation of cytarabine [123]. While promising, the study by Azoulay et al. was limited by its single-arm, single-center design and the inherent diagnostic uncertainty regarding the cause of the respiratory failure [122]. Therefore, those findings need to be validated in larger, ideally randomized clinical trials as high-doses of dexamethasone in already immunocompromised and critically ill patients can potentially be associated with substantial adverse events.

Finally, advances in targeted therapies with the approval of the FLT3 inhibitors gilteritinib and for R/R-AML and midostaurin in the frontline setting in addition to cytarabine-anthracycline induction chemotherapy, respectively, have enabled a personalized approach to the treatment of AML patients [103,104]. As the presence of *FLT3*-ITD mutations has been associated with a higher incidence of hyperleukocytosis, those agents can be a valuable adjunct in those patients. Additionally, advances in supportive care and aggressive management of TLS and DIC with uric acid-lowering agents such as rasburicase, renal replacement therapy, and transfusion of blood products have led to a decrease in the mortality rate during AML induction although specific data from patients with hyperleukocytosis and leukostasis are missing [96,124].

## 7. Conclusions

Hyperleukocytosis in AML is associated with excess mortality and higher rates of leukostasis, DIC, and TLS compared to patients without hyperleukocytosis. On a molecular level the processes leading to hyperleukocytosis and leukostasis are still incompletely understood but the several key targets mediating the interaction between the leukemic blast and endothelial cells in the peripheral circulation continue to be identified. Additionally, translational studies investigating the interaction of AML blasts and the bone marrow microenvironment could lead to a better understanding of the processes underlying leukostasis. However, one needs to be mindful of the limitations of extrapolating those results to the clinical setting. Nonetheless, an understanding of these molecular mechanisms is crucial for the rational development of novel targeted agents with GMI-1271 (uproleselan) and plerixafor currently being studied in clinical trials. Because of the rarity and emergent nature of this presentation, clinical trials specifically enrolling AML patients with hyperleukocytosis have proven to be very difficult to conduct and patients with hyperleukocytosis have either been excluded from recent randomized trials or results have not been reported separately for this high-risk patient population. Especially regarding the management of leukostasis, the evidence base is limited and mixed; although leukapheresis has not been shown to be associated with improved survival in several recent retrospective studies and meta-analyses. While awaiting novel therapies, cytoreduction with intensive chemotherapy, leukapheresis, and/or hydroxyurea as well as excellent supportive care of TLS and DIC remain the mainstay of therapy and physicians need to be mindful of the gaps in the evidence base for these interventions.

## Figures and Tables

**Table 1:** Overview of selected studies identifying disease characteristics associated with hyperleukocytosis among AML patients.

Risk Factor	Patient Population (References)	Risk Factor Associated with Hyperleukocytosis
FAB subtypes M4 and M5	Single center retrospective studies of AML patients with hyperleukocytosis [5,6,8,58]	45–73% of AML patients with hyperleukocytosis with FAB M4/5 [5,6,8,58]
*MLL* gene rearrangements	(1) 11 patients with 11q translocation-associated acute leukemia [59] (2) Single center retrospective review of 52 AML patients with hyperleukocytosis [8]	(1) 8 out of 10 patients with *MLL* gene rearrangement 11q23 presented with hyperleukocytosis [59] (2) 5.8% of patients with hyperleukocytosis with *MLL* gene rearrangements
Presence of selected mutations (e.g., *FLT3*)	(1) Single center retrospective study of 693 de novo AML patients [56] (2) 977 AML patients treated on AML-96 study protocol [15]	(1) Following genes more common in patients with WBC >50,000/µL: *FLT3-*ITD (32.5% vs. 13.9%, *P* < 0.0001), *NPM1* (30% vs. 17.4%, *P <* 0.0001), *DNMT3A* (25.1% vs. 14.2%, *P* = 0.0001), *CEBPA* (23.6% vs. 10.4%, *P* < 0.0001)*, TET2* (21.9% vs. 7.9%, *P* < 0.0001), and *NRAS* mutations (20% vs. 13.4%, *P* = 0.03) (2) Median WBC at presentation: *FLT3*-WT: 10,900/ µL; *FLT3*-ITD: 52,000/µL; *FLT3*-TKD: 40,100/µL; *FLT3*-ITD and *FLT3*-TKD: 50,700/µL

**Table 2:** Overview of recent randomized trials leading to the approval of novel agents and results regarding patients with hyperleukocytosis.

Author [Reference]	Trial Design	Patient Population	Proportion of and Outcomes among Patients with HL
DiNardo et al. [110]	Phase I trial; single arm ivosidenib monotherapy	258 patients with *IDH1* mutations; 179 with R/R-AML	3.5% of patients with WBC ≥ 30,000/µL; outcomes not reported separately; 36.8% with leukocytosis while receiving ivosidenib
Stein et al. [105]	Phase I/II trial; single arm enasidenib monotherapy	239 patients with IDH2-mutated R/R-AML or MDS-RAEB	Median WBC 2600/µL (R: 0.2–88); proportion and outcomes of patients with HL not reported; 17% of patients with worsening non-infectious leukocytosis
Lancet et al. [108]	Phase III randomized trial of CPX-351 vs. standard 7 + 3	309 patients 60–75 years with newly diagnosed secondary AML or AML-MRC	14.4% of patients with WBC ≥ 20,000/µL; OS significantly inferior compared to WBC ≤ 20,000/µL (HR 0.67 (95% CI: 0.45 to 0.98); p = 0.04)
Stone et al. [104]	Phase III randomized trial of midostaurin vs. placebo in addition to standard 7 + 3	717 patients 18 to 59 years of age with *FLT3-*mutated newly diagnosed AML	Median WBC 34,900/µL (R: 0.600–421,800); no impact of higher WBC on OS but adverse impact on EFS (HR: 1.018 [95% CI: 1.001–1.035]; p = 0.04)
Perl et al. [103]	Phase III randomized trial of midostaurin vs. salvage chemotherapy	317 patients with *FLT3*-mutated R/R-AML	No information on WBC or outcomes reported
DiNardo et al.	Phase III randomized trial of azacitidine + venetoclax vs. azacitidine + placebo	431 newly diagnosed AML patients ≥75 years or ineligible for intensive chemotherapy	WBC ≥ 25,000/µL excluded (cytoreduction with hydroxyurea or leukapheresis permitted)
Wei et al. [100]	Phase III randomized trial of low-dose cytarabine + venetoclax vs. low-dose cytarabine + placebo	210 newly diagnosed AML patients ≥75 years or ineligible for intensive chemotherapy	WBC ≥ 25,000/µL excluded (cytoreduction with hydroxyurea or leukapheresis permitted)
Castaigne et al. [106]	Phase III randomized trial of gemtuzumab ozogamicin + standard 7 + 3 vs. standard 7 + 3 alone	210 newly diagnosed AML patients 50–70 years	Median WBC 5900/µL (IQR: 2.100–29,100); proportion and outcomes of patients with HL not reported
Cortes et al. [107]	Phase II randomized trial of glasdegib + low-dose cytarabine vs. low-dose cytarabine alone	132 newly diagnosed AML and high-risk MDS patients ineligible for intensive chemotherapy	WBC ≥ 30,000/µL excluded (cytoreduction with hydroxyurea or leukapheresis permitted)

AML—acute myeloid leukemia; AML-MRC—AML with myelodysplasia-related changes; EFS—event-free survival; HL—hyperleukocytosis; HR—hazard ratio; IQR—interquartile range; MDS—myelodysplastic syndrome; MDS-RAEB—MDS—refractory anemia with excess blasts; OS—overall survival; R—range; R/R—relapsed/refractory; WBC—white blood cell count.
